# Are miRNAs Dynamic Biomarkers in Keratoconus? A Review of the Literature

**DOI:** 10.3390/genes13040588

**Published:** 2022-03-25

**Authors:** Spela Stunf Pukl

**Affiliations:** 1 Medical Faculty, University of Ljubljana, 1000 Ljubljana, Slovenia; spela.stunf@siol.net; Tel.: +386-41-382-487; 2 Eye Hospital, University Clinical Center Ljubljana, 1000 Ljubljana, Slovenia

**Keywords:** biomarkers, keratoconus, miRNA

## Abstract

Aim: A review of miRNA (microRNA) profiling studies in keratoconus. Methods: Literature search strategy—PubMed central database, using miRNA or microRNA and keratoconus as keywords. Results: Eleven experimental or clinical studies on humans regarding miRNA and keratoconus, published in English between 2009 and 2020 were retrieved. Conclusion: The publications regarding the role of miRNAs in keratoconus are scarce and diverse but provide some valuable information about potential new mechanisms of keratoconus development and progression. The cornea expresses almost 300 different miRNAs, 18 of which are specific, and miR-184 is by far the most abundant, with expression restricted to central basal and suprabasal epithelial cells. Mutations in the seed region of *MIR184* were proved to be rare and nonspecific in patients with isolated keratoconus. Overall, in keratoconus, a total of 29 miRNAs were upregulated, and 11 were downregulated. It appeared that miR-143-3p, miR-182-5p, and miR-92a-3p were highly expressed, while the miRNAs connected to cell–cell junction, cell division, and motor activity were downregulated. In less advanced forms, altered expression of four miRNAs—miR-151a-3p, miR-194-5p, miR-195-5p, miR-185-5p—was proved in the cone epithelium; in contrast, in advanced keratoconus, the expression of miR-151a-3p and miR-194-5p remained altered, changes in the expression of miR-195 and miR-185 were not reported, and the expression of miR-138-5p, miR-146b-5p, miR-28-5p, and miR-181a-2-3p was also altered in the corneal epithelium. Keratoconus is a dynamic process of corneal stromal thinning that might result from a dynamic miRNA expression in the corneal epithelium exposed to environmental and behavioral factors causing repetitive traumas. Further experimental studies are needed to prove this hypothesis.

## 1. Introduction

**Figure 1:**
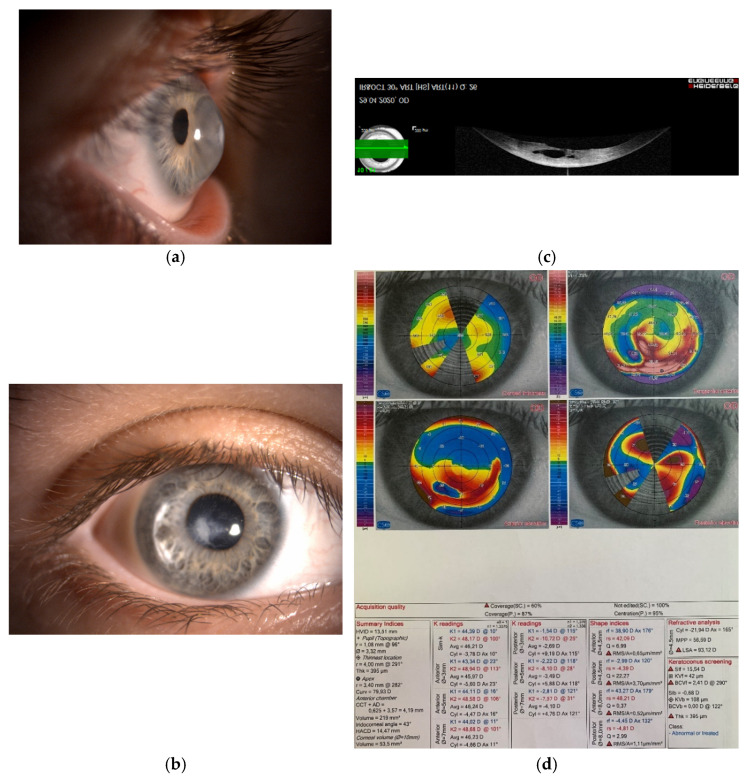
(**a**,**b**) Photography of the right cornea in a 45-year-old female patient with advanced keratoconus and visual acuity of finger counting without correction. The patient was intolerant to the rigid contact lenses. (**c**) Anterior segment optical coherence topography of the same cornea. Due to profound corneal protrusion, a break in the posterior cornea caused swelling of the stroma—called corneal hydrops. (**d**) Severe changes of the corneal topography, which were the underlying cause of severe visual deterioration.

Keratoconus—a noninflammatory progressive corneal disorder characterized by thinning, protrusion, scarring of the corneal tissue, and consequent vision distortion of various severity (Figure 1)—is an etiologically complex multifactorial disease, involving multiple genes, rather than mutation in a single gene, as well as behavioral, lifestyle, and environment factors [1].

**Figure 2:**
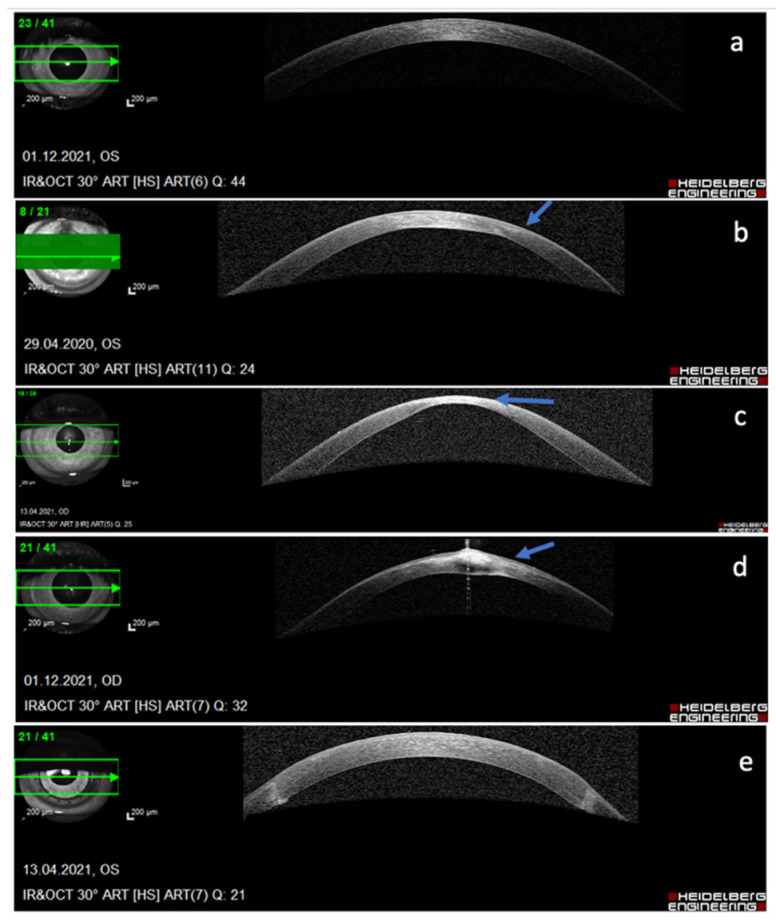
Different stages of keratoconus as demonstrated by anterior segment optical coherence topography (AS OCT) of the cornea: (**a**) sub-clinical stage, no changes seen on AS OCT, (**b**) thinning of the stroma at the cone with compensatory epithelial thickening (arrow), (**c**) significant thinning and bulging of the cone (arrow), (**d**) bulging, hydrops, and fibrosis of the cone (arrow), (**e**) end-stage keratoconus treated with penetrating corneal transplantation.

As such, the onset of the first symptoms usually lags behind the subclinical tissue changes. The aim is to diagnose the early stages of the disease, when noninvasive or minimally invasive interventions can restore normal vision, and prevent progression to severe stages, that require corneal transplantation (Figure 2). The specific topographic patterns of early keratoconus (Figure 1) can be found with a meticulous exam in young patients, for example, screening candidates before refractive laser surgery. When and why the first genetic changes appear in patients with keratoconus is still a matter of speculation and research. However, environmental and the behavior factors such as eye rubbing were recognized as triggering factors of the disease and of its progression from subclinical to advanced forms (Figure 2), typically occurring in young adults.

Epigenetics is an intrinsic mechanism that fills in the gap between genes and environmental factors causing diseases. It represents heritable changes in gene expression not due to changes in the DNA sequence [2]. Genetic transfer to the next generation is not explained by a change in the DNA sequence. i.e., mutations, but rather by reversible epigenetic mechanisms, i.e., epimutations, such as, for example, DNA methylation, histone and non-histone post-translational modifications, and non-coding-RNA modifications. This review focuses on keratoconus and miRNAs as possible biomarkers of this disease, as post-transcriptional regulation by miRNAs has been proved to be an important mechanism of post-transcriptional regulation in other corneal pathologies [3].

## 2. Materials and Methods

A literature search was performed in PubMed central database, using miRNA or microRNA and keratoconus as keywords.

## 3. Results

### 3.1. MicroRNAs

MicroRNAs (miRNAs) are small endogenous noncoding RNAs of approximately 22 nucleotides. They regulate gene expression through targeted binding to messenger RNA (mRNA), which in most cases causes RNA silencing [4]. There are several other mechanisms of action of miRNAs, such as activation of translation or regulation of transcription, while extracellular miRNAs can also function as chemical messengers in cell-to-cell communication [5]. At least 2000 miRNAs have been identified in humans [6,7]. miRNAs are present in cells and also as circulating extracellular miRNAs in different body fluids (blood, cerebrospinal fluid, aqueous humor, tears). MiRNAs are recognized as evolutionarily conserved and have important biological functions regulating the basic cellular functions, such as cellular proliferation, differentiation, and death. MiRNAs regulate more than 30% of the human genome, and they do so primarily by suppressing gene expression. The 22-nucleotide RNA is converted to a primary miRNA by RNA polymerase II and to a mature RNA by the ribonuclease III Drosha. miRNAs target their downstream mRNAs by base-pairing to their target sites with sequence complementarity, mainly in the 3′ untranslated region (UTR). Once bound, the participating miRNA induces a silencing complex (RISC). The bound mRNA is consequently either degraded or inhibited, and gene transcription is surpassed. The mRNA is either cleaved into two pieces, destabilized through shortening of its poly(A) tail, or less efficiently translated into proteins by ribosomes [8].

miRNAs are able to bind and manipulate the expression of multiple targets, which allows broadly varying gene expression, in a manner either beneficial or detrimental to health [9,10]. miRNAs are expressed in a tissue-specific and developmental stage-specific way [11].

Mutations in miRNAs and/or the target sites in the transcripts of their downstream target genes and dysregulation of miRNA biogenesis can result in a wide variety of diseases, including cancer [6,12,13], atherosclerosis [14], asthma [15], neurodegenerative diseases [16]. miRNAs were recognized as potential biomarkers of different diseases as well as possible treatment targets.

### 3.2. MicroRNA and the Eye

In the past decade, investigations of miRNAs expression in different un-diseased ocular tissues were carried out, and the miRNA transcriptomes of the retina, lens, and cornea have been established [17]. Many miRNAs show unique tissue-specific and developmental stage-specific expression patterns, suggesting potential unique functions in the retina and other ocular tissues. 

Profiling miRNA expression in human ocular tissues using second-generation miRNA sequencing (miRNA-Seq) was performed to determine the entire spectrum of miRNAs present in the heathy human cornea [18]. By studying un-diseased ocular tissues, it was found that the most abundant miRNAs are miR-143-3p, miR-184, miR-26a-5p, and miR-204-5p [19]. Additional studies proved that 11 of 378 miRNAs were highly expressed and represented 80% of total number of mean normalized reads. As a rule, a few miRNAs represent most of the total miRNAs present in a specific cell type. Large amounts of these miRNAs indicates that they are important to the function of these tissues. Only miRNAs present in a sufficient concentration have an impact on cell regulation [20].

Among the pathological processes of the anterior part of the eye that were recognized to be modified by miRNAs, neovascularization of the cornea is promoted by miR-132, miR-155, miR-206, and miR-21 and repressed by miR-184 [21]. Modulation of the miRNA network was also suggested as a possible treatment target for corneal neovascularization [22]. A number of different corneal infections, from ocular trachoma, to bacterial, fungal or viral keratitis, are also under the influence of miRNAs, as miRNAs regulate innate and adaptive immunity [23].

### 3.3. MicroRNA and the Cornea

Few studies addressed the expression and function of miRNAs in non-diseased cornea. Ryan et.al. analyzed corneal miRNAs in a mouse model and proved highly specific expression of miR-184 in the corneal epithelium, specific and cornea-restricted expression of miR-204 and miR-31, and significant expression, though not restricted to the cornea, of miR-205 and let-7. miR-184 was found in basal and supra basal epithelial cells [24].

A study on cultured cell lines that compared the expression of miRNAs in the corneal epithelial stem cells to the expression of miRNAs in differentiated corneal epithelial cells, identified 62 miRNAs in stem cells and 611 miRNAs in differentiated corneal epithelial cells. Six miRNAs were found to be significantly upregulated in stem cells, i.e., miR-21-5p, miR-3168, miR-143-3p, miR-10a-5p, miR-150-5p, and miR-1910-5p. Expression of miR-143-3p was exclusive to clusters of limbal basal epithelial cells. In differentiated corneal epithelial cells, the most abundantly expressed miRNAs were miR-181a-5b, miR-184, miR-let-7a-5b, miR-92a-3a, miR-7b-5b, miR-205-5b, miR-204-5b, miR-26a-5b, miR-4485-3b, miR-181b-5 b [25]. 

By analyzing the corneal tissue separated from the collective eye tissue, Drewry M. et.al. found that miR-184 was the most abundant miRNAs in the cornea [19]. The cornea was found to express altogether 297 miRNAs, 18 of which appeared expressed uniquely in the cornea. To demonstrate a possible tissue specificity of these miRNAs, miRTarBase, (accessed on 2 July 2016), was used to analyze the gene targets of these miRNAs. Twenty-eight gene targets were found to be shared by 11 of these 18 miRNAs. Of the remaining seven miRNAs, four had no high-confidence targets, and three shared no gene targets with the other uniquely expressed miRNAs. Ten of these 28 shared gene targets were regulated primarily by the uniquely expressed miRNAs *BAP1* (100%), *DLX4* (100%), *IL24* (100%), *INPPL1* (100%), *RERE* (100%), *SIP1* (100%), *WASF3* (75%), *ZEB1* (88%), *ZEB2* (88%), and *FPM 2* (100%). Five of the corneal uniquely expressed miRNAs, i.e., miR-141-3p, miR-200a-3p, miR-200b-3p, miR-200c-3p, and miR-429, were found to be the most similar with respect to shared gene targets. They were found to share 47%, 77%, 80%, 50%, and 88% of their gene targets with the other uniquely expressed miRNAs, respectively [19].

#### miR-184 and miR-205 in the Cornea

Abu-Amero et al. [26] studied the expression of mature miRNAs sequenced from post-mortem human ocular samples. In the corneal tissue, both miR-184 and its competitor miR-205 were the most abundantly and highly specifically expressed miRNAs, with a mean of 45,039 reads per million sequencing reads for miR-184, and a mean of 2867 reads per million sequencing reads for miR-205. The expression of miR-184 was almost 15-fold higher than the expression of miR-205 in the human cornea [26].

miR-184 (encoded by *MIR184* (MIM 613146)) is in fact specific to and the most abundant miRNA in the corneal epithelium. It is expressed in the central corneal epithelial basal and suprabasal cells. Interestingly, the expression of miR-184 is restricted to the central cornea and is absent in the limbal epithelium, an area enriched in corneal epithelial stem cells [27]. The other most important miRNA in the cornea is miR-205. It is not specific to this tissue but is rather broadly expressed throughout all viable cell layers in most stratified squamous epithelia of the eye, including corneal, limbal, and conjunctival epithelia [28]. The corneal epithelium is, however, unique for its distinct as well as overlapping expression of miR-184 and miR-205 [29].

miR-184 has a unique role related to the regulation of proliferation versus differentiation in corneal epithelial cells. Its expression is independent of the proliferative stage of the epithelium, but it does delineate the differentiated phenotype of the epithelium in a complex cross-talk with miR-205. miR-184 prevents the knockdown of INPPL1 (SHIP) and ITGB4. The cross-talk between miRNA-184 and miRNA-205 is the first example in a vertebrate system of an miRNA abrogating the inhibitory function of another [24,29].

Using human epithelial cell lines and primary human corneal and epidermal keratinocytes, Yu et al. [30] in fact showed that MIR184 interferes with the ability of MIR205 (613147) to downregulate the expression of SHIP2 (INPPL1; 600829)—a lipid phosphatase that functions in the AKT signaling pathway [30].

A synthetic antagomir of MIR205 or the ectopic expression of miR-184 induced SHIP2 expression in keratinocytes, with coordinated damping of AKT signaling and increased apoptosis. SHIP2 mRNA is a target of miR-205 trough translational repression, while miR-184 antagonizes the inhibitory effect of miR-205 on SHIP2 levels, thereby maintaining high SHIP2 levels [30].

The negative regulation of miR-205 by miR-184 is unique in the cornea, and the corneal epithelium is the only known epithelium that exhibits overlapping expression of miR-184 and miR-205 [30]. Examination of the 3’ UTR of SHIP2 revealed overlapping binding sites for MIR184 and MIR205. Co-expression of MIR184 with MIR205 reversed MIR205-induced inhibition of a reporter gene containing the SHIP2 3’ UTR. miRNA184 had no direct effect on SHIP2 expression but instead interfered with miRNA205 binding to the 3’ UTR of SHIP2 [30]. 

miR-184 has a broad network of target genes connected to ion transport, cell migration, differentiation, regulation of transcription, actin organization, Wnt signaling pathway, and cell proliferation [31]. The expression of as many as 1000 genes might be regulated by miR-184, independently or in competition with other miRNAs, which has a complex effect on a large number of proteins. The harmful effects of mutant miR-184 might be mediated through proteins other than SHIP2 and ITGB4. Among the other important predicted targets of miR-184 are a major lens transcription factor (*FOXE3* (MIM 601094)) and a major intrinsic protein of eye lens fiber (*MIP* (MIM 154050)). Mutations in both genes cause lens abnormalities in humans [32].

Normal miRNA expression in the cornea is known to be connected to normal corneal epithelial regeneration [33] and normal corneal wound healing [34], while abnormal miRNA expression was found in Sjogren syndrome, ocular surface neoplasia, corneal dystrophies and keratoconus [18], and in corneal wound healing in diabetics [35].

### 3.4. Keratoconus and MicroRNA 

The tissue-specific expression of miR-184 is of prime importance, when discussing the phenotypic effects of a mutation. Within the cornea, the expression of miR-184 is restricted to central basal and supra-basal epithelial cells, under which stromal thinning occurs in keratoconus. Pathologically, keratoconus is thought to result from the dysregulation of epithelial cells’ apoptosis. Histological studies also proved changes in the epithelium before any clinical manifestation of the disease [36]. Namely, thinner loosely packed cells and defects or breaks in epithelial basement membrane Bowman’s layer were found on the histological level [37].

In contrast to protein-coding genes, sequence variants in mature miRNAs are extremely rare in germ-line cells. This is possibly due to the extreme conservation and importance of mature miRNAs, as well as to the really small size of these molecules (18–25 base pairs). Known variants are limited to the polymorphism in MIR125a [38] and the mutation in MIR96 causing autosomal-dominant progressive hearing loss [39,40].

#### 3.4.1. miRNA-184 in Keratoconus

Mutations in MIR184 have been studied in patients with keratoconus. Hughes et al. [28] found a heterozygous C-to-T transition (r.57C > T) within the *MIR184* seed region in a Northern Irish family, in which 18 individuals from 3 generations were affected with keratoconus associated with cataract (Table 1) [28]. The seed sequence is a conserved heptametrical sequence, mostly situated at positions 2–7 from the miRNA 5′ end. Even though base pairing of miRNA and its target mRNA is not perfect, the “seed sequence” has to be perfectly complementary to the target sequence of mRNA [32].

Lechner et al. [41] identified two heterozygous substitution mutations in the seed region of *MIR184* (+3A > G and +8C > A) in two patients with sporadic keratoconus not associated with other ocular comorbidities; one of the two patients had, however, a concurrent cataract (Table 1) [41]. This represents 0.25% of 780 reported screened cases of sporadic isolated keratoconus. This indicates that this sequence variant may account for a very small percentage of isolated keratoconus cases [41].

Bykhovsaya et al. [42] also identified a c.57C > T mutation in *MIR184* affecting five generations of a family from Galicia, Spain, with keratoconus and congenital cataracts. Individuals with a c.57C > T mutation in *MIR184* displayed varying corneal abnormalities, from severe keratoconus to non-ectatic corneal thinning (Table 1) [42].

Abu-Amero et al. [26] could not identify any point mutations, small insertions, or small deletions in the seed region of *MIR184* in any of the 134 Arabic patients with familiar or sporadic isolated keratoconus. They concluded that mutations in the seed region of *MIR184* are rare in patients with isolated keratoconus and may be more relevant to cases of keratoconus associated with other ocular abnormalities [26].

A substitution mutation within the seed region of *MIR184* has been however mapped in patients with other corneal pathologies or abnormalities in the anterior segment of the eye, such as endothelial dystrophy, iris hypoplasia, congenital cataract, and stromal thinning not associated with keratoconus (EDICT syndrome) [43,44].

**Table 1:** miRNAs in keratoconus.

	Type of Mutation Found	Circumstances	Authors
Mutations in the MIR184 seed region	heterozygous C-to-T transition (r.57C > T) in the *MIR184* seed region	Irish family. 18 individuals from three generations were affected. Keratoconus associated with cataract	Hughes et al. [32]
	Two heterozygous substitutions (+3A > G and +8C > A) in the *MIR184* seed region	Two patients. Sporadic keratoconus, no other ocular comorbidities, one had concurrent cataract	Lechner et al. [41]
	heterozygous C-to-T transition (c.57C > T) in the *MIR184* seed region	Spanish family from Galicia. five generations. Congenital cataracts, varying corneal abnormalities, from severe keratoconus to non-ectatic corneal thinning.	Bykhovsaya et al. [42]
Other miRNAs, altered expression	upregulated, highly expressed: miR-143-3p miR-182-5p miR-92a-3p	Keratoconus corneal tissue.	Drewry M et al. [19]
	altered expression of six miRNAs: miR-151a-3p miR-138-5p miR-146b-5p miR-194-5p, miR-28-5p miR-181a-2-3p altered expression of four miRNAs: miR-151a-3p miR-195-5p miR-185-5p miR-194-5p	Corneas obtained from keratoconus transplant surgeries. Samples of epithelium obtained by means of impression cytology	Wang YM et al. [37]
	3 miRNAs upregulated: miR-128, miR-32, miR-221 4 miRNAs downregulated: miR-181a, miR-222, miR-98, miR-301a	Keratoconus-associated RNA regulatory network.	Tyan R et al. [45]

#### 3.4.2. Other miRNAs in Keratoconus

For keratoconus, the highly expressed regulators were proved to be miR-143-3p, miR-182-5p, and miR-92a-3p. All of these highly expressed disease-associated miRNAs are included in the top 18 most abundant miRNAs in collective ocular tissues (Table 1) [19].

A study compared the corneal epithelium of 27 keratoconus patients to that of 26 controls by histology, miRNA microarrays, gene ontology, pathways analyses, TaqMan PCR (polymerase chain reaction), immunofluorescence, and immunoblotting analyses. The keratoconus samples were obtained either at the time of keratoconus corneal transplant surgery or by means of epithelial impression cytology. The histological changes in keratoconus included thinner loosely packed epithelial cells and defects or breaks in the Bowman’s layer, which serves as an epithelial basement membrane. [37]. Twelve miRNAs were significantly downregulated. Their predicted target genes participate in cell junction, cell division, and cell motor activity. The involved pathway was proved to be the Syndectan signaling pathway. In keratoconus corneas obtained from keratoconus transplant surgeries, the epithelium showed altered expression of six miRNAs, i.e., miR-151a-3p, miR-138-5p, miR-146b-5p, miR-194-5p, miR-28-5p, and miR-181a-2-3p, while the keratoconus corneal epithelium obtained by impression cytology showed altered expression of four miRNAs, i.e., miR-151a-3p, miR-195-5p, miR-185-5p, and miR-194-5p (Table 1) [37].

A study by Tian et al. [45] analyzed post-transcriptional regulation in keratoconus at the level of miRNAs and long noncoding RNAs (lncRNAs). lncRNA can regulate miRNA activity trough sponge absorption and competitive mRNA binding. There was a difference in the expression in 40 miRNAs (29 upregulated and 11 downregulated, including miR-181a, miR-222, miR-98, miR-301a), 282 lncRNAs (192 upregulated and 90 downregulated), and 910 mRNAs (554 upregulated and 356 downregulated) when comparing keratoconus to myopic control samples (Table 1) [45].

A total of 34 functional terms and 9 pathways were enriched for the mRNAs [33]. In conclusion, the study claimed that six mRNAs (including *PPARG*, *HLA-B*, *COL4A1*, and *COL4A2*), five miRNAs (including upregulated miR-128, miR-32, miR-221 and downregulated miR-181a, miR-222, miR-98, miR-301a), nine lncRNAs (including *XIST*—X-inactive specific transcript—a non-coding RNA on the X chromosome that acts as a major effector of the X-inactivation process), and the *XIST-miR-181a-COL4A1* axis are involved in the keratoconus-associated RNA regulatory network [45]. The included pathways participate in focal adhesions, cytokine–cytokine receptor interactions, chemokine signaling pathways, ECM–receptor interactions, type I diabetes mellitus, pathways in cancer, cytosolic DNA-sensing pathways, vascular smooth muscle contraction [45].

## 4. Discussion

There are still many open questions regarding the ethology, onset, and progression of keratoconus. For patients with keratoconus, the possibility of establishing a diagnosis in the early disease stages and immediately after the detection of signs of progression is very important. Besides, identifying biomarkers of early-stage keratoconus and progressive keratoconus would be a potential clinical advancement.

This review of the published literature on miRNAs in keratoconus proved no unique biomarker among the disease-specific miRNAs. 

Mutations in the *MIR184* gene were mostly confirmed in keratoconus in association with other abnormalities of the anterior segment. In cases of isolated keratoconus, mutations in *MIR184* were reported to be rare [26,32,41,42]. Up- or downregulation of several other miRNAs [19,37,45] was found in keratoconus, but the expression patterns were not connected to the stage of keratoconus at the time of sampling. In studies where corneas were obtained at the time of keratoconus corneal transplant surgery [37], the disease was probably already in the advanced stage. Sampling with the aid of impression cytology is a minimally invasive method and can be performed in patients with suspected keratoconus or early disease.

One of the basic pathological mechanisms of keratoconus development is believed to be the dysregulation of apoptosis [46]. In the cornea, it was proved that miR-184 and miR-205 cross-talk is of major importance in the regulation of apoptosis. Histologically, signs of dysregulation of apoptosis appear as changes in the corneal epithelium, such as the presence of loosely packed epithelial cells, thinning of the epithelium, scaring of the epithelium, vacuolar changes of epithelial cells [23,37,47], and can be found before the disease is clinically manifested. In preclinical stages, changes in the relationship between miR-184 and miR-205 in epithelial samples could have a biomarker role.

One of the most important behaviors causing keratoconus was recognized to be eye rubbing. Review studies proved a consistent association of eye rubbing and keratoconus, the degree of the effect of eye rubbing being dependent on the period and force of this action [48,49]. Many different processes take place during and after the act of eye rubbing. There is an increase of the temperature in the epithelium, an increase in the concentration of inflammatory molecules in the precorneal tear film, as well as subsequent epithelial thinning, mechanical trauma to the epithelium, and other changes [48].

Elevated temperature per se influences changes in miRNA expression, as proved in studies [50,51]. An increased inflammatory response in tears increases the expression of miR-184 through IL-22 and downregulates argonaut 2. Argonaut 2 is an essential protein of the RNA-induced silencing complex RISC. Downregulation of argonaut 2 causes an imbalance in the cross-talk between miR-184 to miR-205, leading to increased apoptosis and cell death [52]. Repetitive epithelial injury results in increased IL-1 α release from the epithelium to the stroma, leading to keratocyte activation, MMP2 (matrix metalloproteinse-2) secretion, and keratocyte death. The release of TGF-beta2 from the epithelium to the stroma, on the other hand, transforms keratocytes to myofibroblasts, which than recreate the ECM (extracellular matrix) [53].

A constant trauma fires the process of corneal wound healing, with natural powerful changes in the regulation of gene expression. Epithelial defects are healed by the migration and proliferation of epithelial cells. In the phases of migration and proliferation, increased expression of miR-205 occurs, decreasing the levels of SHIP, resulting in decreases epithelial cells’ apoptosis and increased epithelial as well as keratocyte migration trough the Act – protein kinase B signaling pathway [54].

microRNAs were proved to have tissue-specific as well as event- or pathological state-dependent dynamic expression in animals [55]. A dynamic expression of miRNAs was also proved as a response to hypoxia in fish [56], as well as in heart regeneration in zebrafish [57] and in the development of immune cells [58].

When a non-diseased corneal epithelium is wounded, the most abundantly expressed corneal miRNA, i.e., miRNA-184, is downregulated in re-epithelializing epithelial cells. It is also known that this process results in unopposed effects of miRNA-205, decreased levels of SHIP2 (INPLL1), and lack of apoptosis or cell death. In corneas with active progressing keratoconus, where repetitive epithelial traumas occur due to eye rubbing, an anterior-segment—optical coherence tomography (AS-OCT) study revealed epithelial irregularity with inferior relative thickening corresponding to the steep area measured by the Oculyzer tomographer [59]. Changed profiles or miRNAs connected to wound healing as well as an unbalance of miR-184 to miR-205 levels in epithelial samples could serve as a sign of active disease.

The majority of visual problems in established and late stages of the disease occur due to changes in the corneal stroma. The corneal stroma undergoes thinning, ectasia, and scaring, resulting in changed tectonic and optic characteristics. The epithelial basement membrane and Bowman’s layer in keratoconus corneas present defects, breaks, and changes in their composition, which cannot be explained by scarring alone. A process similar to wound healing was suggested to largely contribute to the differences seen in keratoconus corneas [60].

During corneal epithelial wound healing, the basement membrane, when damaged or even only denudated and exposed for longer than 24 h, undergoes degradation and subsequent reassembly [53]. It was suggested that the activation only of the denudated and exposed basement membrane is the result of transient amplifying cells (TACs) activity. They heal the wound from the corneal periphery to the center by centripetal movements similar to standard epithelial renewal [61]. TACs also remodel the central basement membrane for the proper movement of epithelial cells and healing without erosions. 

When the epithelial basement membrane integrity is compromised during wounding, there can be consequences for the healing of the entire cornea, rather than of the epithelium alone. In normal conditions, the epithelial basement membrane controls the availability of epithelium-derived growth factors and cytokines (transforming growth factor β-1—TGF-β1—and PDGF) to stromal cells. Communication occurs also in the opposite direction, as stromal-derived growth factors (keratinocyte growth factor—KGF) are delivered to the epithelium. Injury to the epithelial basement membrane compromises the barrier function, allowing the penetration of cytokines from the epithelium to the stroma and back and the access of stromal cells to epithelial cytokines and vice versa. This is thought to cause keratocyte apoptosis by IL-1 and keratocyte-to-myofibroblast transformation due to TGF-β and PDGF access to the stroma [62,63,64]. Declined levels of TGF-β1 and PDGF in the stroma due to the reassembly of the epithelial basement membrane leads to the apoptosis of stromal myofibroblasts, which depend on TGF-β1 for their survival [64,65,66]. 

In the cornea, some miRNAs display topographical expression differences between central part, limbus, and adjacent conjunctiva [11,29,33,67,68]. Several miRNAs with preferential limbal epithelial localization appear to play a role in delayed wound healing in diabetic corneas [35,69].

The emerging evidence suggests the importance of miRNAs in many phases of the corneal epithelial wound healing process, where some miRNAs promote healing, while others inhibit it.

During migration and proliferation of corneal epithelial cells at the wound edge, upregulated miR-205 targets SH2-containing phosphoinositide-5-phosphatase (SHIP2), which in turn affects Akt signaling and increases migration [54]. The upregulated miR-205 can additionally promote motility of epithelial cells through modification of F-actin organization [54]. It has been further shown that miR-205 facilitates the wound healing process through inhibition of another target gene, coding for KCNJ10 channel, in human corneal epithelial cells [70]. In skin keratinocytes, growth arrest at the final steps of re-epithelialization is regulated by miR-483-3p acting on MK2 kinase and transcription factor YAP1 [71]. miR-204, which targets the *SIRT1* gene, was shown to impair the cell cycle progression of corneal epithelial cells in DM2 diabetic Akita mice [72] and to inhibit cell migration [34]. miRNA-204 was dramatically downregulated in wounded epithelium. Transfection of cultured cell with miR-204 caused cell arrest in G1 phase, suppressed the regenerative response, and inhibited cell migration.

Abnormal healing in diabetic corneas was connected to dysregulation of miR-146a and miR-424 [35]. miR-146a plays a major role as a modulator of the innate immune and inflammatory responses and wound inflammation and may repress proinflammatory genes within the wound [73].

These recent data attest to the significance of miRNAs in regulating corneal wound closure and provide novel insights into mechanisms of the wound healing process. Dysregulation of miR-146 was proved in keratoconus corneas obtained at the time of the surgery, which is undertaken in cases of advanced keratoconus, but not from keratoconus corneal epithelia obtained by means of impression cytology, a minimally invasive method for nonsurgical keratoconus cases [37]. This may represent dynamic changes of miRNA expression connected to the dynamic process of keratoconus development and progression.

The miRNAs connected to reactions of the stroma during wound healing could be further studied as biomarkers of keratoconus progression.

Keratoconus affects the young active population, and knowledge about early and effective diagnostics as well as successful halting of keratoconus progression with corneal crosslinking would have an immense impact on patients’ life. There are many pathogenic theories, and genetic predisposition has a role. Epigenetic studies in keratoconus identified certain markers of the disease; however, the information is still incomplete. Further studies are needed to find a clinically applicable method of corneal tissue sampling and identify useful biomarkers of early and progressive disease.
